# A Case Report of Native Coronary Artery Thrombosis With Heparin-Induced Thrombocytopenia

**DOI:** 10.7759/cureus.39220

**Published:** 2023-05-19

**Authors:** Aung Hein, Su M Wai

**Affiliations:** 1 Department of Cardiology, University Hospitals Dorset, Bournemouth, GBR

**Keywords:** heparin-induced thrombocytopenia (hit), oral anticoagulant, anticoagulant, immune mediated reaction, myocardial infarction, stemi, coronary artery thrombosis, lower molecular weight heparin, 4ht score, acute coronary syndrome (acs)

## Abstract

Heparin is an anticoagulant which has been widely used in various clinical settings, from thromboprophylaxis to the treatment of thromboembolism. Heparin-induced thrombocytopenia (HIT) is a rare medical condition with severe complications if unrecognised, and it carries significant risks of co-morbidities and mortality. The incidence of HIT is relatively less common in low molecular weight heparin. HIT is more common in the venous system than the arterial circulatory system, and it is rare to see multi-vessel coronary artery thrombosis due to HIT. We hereby report a case of multi-vessel coronary thrombosis secondary to low molecular weight HIT, presenting as a case of ST-segment elevation myocardial infarction. We learned from the case that low molecular weight heparin can cause thrombosis secondary to HIT and HIT could be one of the differential diagnoses in those presenting with ST-elevation myocardial infarct and recent exposure to low molecular weight heparin.

## Introduction

Heparin-induced thrombocytopenia (HIT) is an immune-mediated phenomenon in which antibodies are directed against the heparin/platelet factor 4 (PF4) complex, and the complex binds to the platelet surface, thereby activating and aggregating platelets. Activated platelets release further PF4 which serves as a binding site for heparin to form the heparin/PF4 complex [1]. The cycle with an increased level of activated platelets may result in the activation of the coagulation cascade and increased tissue factor expression [2]. These events subsequently lead to thrombus formation with increased platelet consumption, and both arterial and venous systems could suffer from thromboembolic complications, while the latter has a higher incidence [3]. Peripheral arterial circulation seems to be more susceptible to arterial complications, while we see central arterial complications such as stroke and myocardial infarction less commonly [4]. Coronary artery thrombosis in the native coronary artery is a rare situation, particularly in the case of HIT after exposure to low molecular weight heparin. Those receiving low molecular weight heparin, such as enoxaparin, have a lower incidence with an absolute risk of 0.2 while unfractionated heparin may have an absolute risk of 2.6 [5]. Gender plays a role in the risk of HIT development, and women have a 1.5 to 2-fold increased risk compared to men [6]. HIT can be divided into type I and type II, where the latter has more severe complications. Diagnosis of HIT still poses a challenge to clinicians as it needs a degree of suspicion for those presenting with thromboembolic complications. The "4Ts" score is a scoring system readily available for clinicians and a reliable tool. Thromboembolic complications of HIT can be life-threatening as they could present as massive pulmonary embolism [7] or myocardial infarction [8]. A sudden drop in platelet with recent exposure to heparin should generate a high degree of suspicion of HIT for the clinicians.

## Case presentation

**Figure 1:**
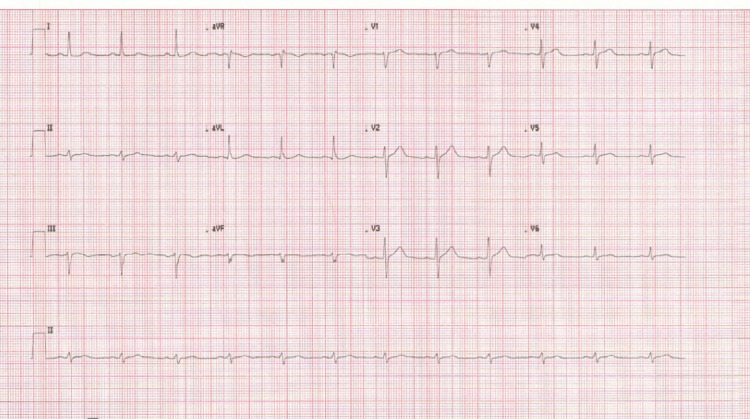
ECG Normal sinus rhythm without ischaemic changes visible on ECG. ECG: electrocardiogram

**Figure 2:**
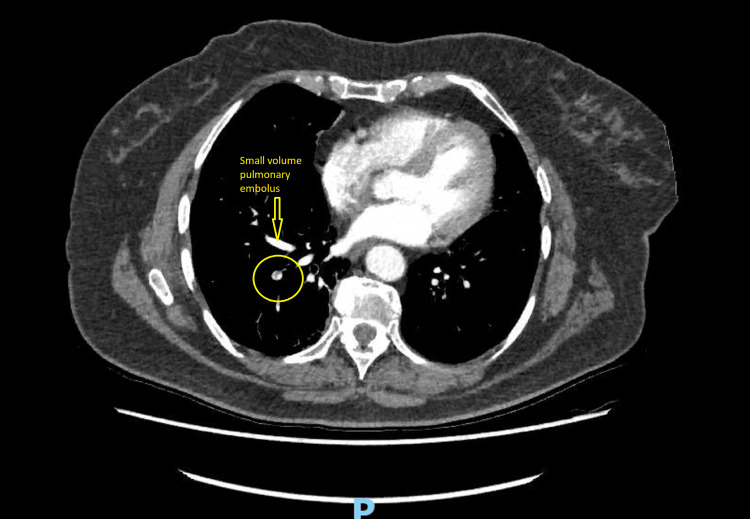
CT pulmonary angiogram CT pulmonary angiogram with small pulmonary embolus (CT was performed as a routine follow-up by the oncology team). CT: computed tomography

An 83-year-old female patient presented with central crushing chest pain to a district general hospital. On arrival, she was diaphoretic, with chest heaviness radiating to her shoulders. She also had lethargy, fever, headache, diarrhoea and vomiting. Her Glasgow Coma Scale (GCS) was 15/15 with eye 4/4, motor 6/6 and verbal 5/5. She was peripherally perfusing well with capillary refill time was less than two seconds, and she did not look dehydrated. Her initial blood gas showed that she was not acidotic, and there was a very mild increase in lactate (Table 1). She had normal first and second heart sounds with no murmur. There were no crackles heard on lung auscultation bilaterally. There was no pitting oedema as well. Her abdominal examination was normal. Her observations were blood pressure of 162/80 mmHg, heart rate of 82 beats per minute, respiratory rate of 16 per minute, oxygen saturation of 95% on room air and temperature of 38.4 degrees Celsius. She had a past medical history of caecal cancer for which she had surgical resection of large bowel two years ago, hypertension, chronic obstructive pulmonary disease and tuberculosis as a child. In recent months, she was diagnosed with recurrent bowel cancer and was on the palliative chemotherapeutic agent capecitabine. She lived alone and was usually independent without any care or support. She was given intravenous fluids and antibiotics to cover possible intra-abdominal infection, given recent chemotherapy, after which the blood tests were sent for further evaluation. Electrocardiogram (ECG) on arrival at the emergency department was in sinus rhythm without any ischemic changes (Figure 1). Ten days before this event happened, she had a routine check-up under oncology for her bowel cancer, and there was an incidental finding of small volume pulmonary embolus (Figure 2) for which she was started on low molecular weight heparin (Dalteparin).

**Table 1:** Venous blood gas pO2: partial pressure of oxygen; pCO2: partial pressure of carbon dioxide

Parameters	Values	Reference range
pH	7.35	7.350-7.450
pO2	7.05	10.0-13.0 kPa
pCO2	5.59	4.50-6.00 kPa
Haemoglobin	148	115-174 g/l
Sodium	138	135-145 mmol/l
Calcium	1.26	1.12-1.32 mmol/l
Potassium	3.8	3.5-4.5 mmol/l
Chloride	104	98-107 mmol/l
Glucose	9.0	3.9-8.0 mmol/l
Lactate	2.5	0.4-2.2 mmol/l
Bicarbonate	22.2	23-29 mmol/l

**Figure 3:**
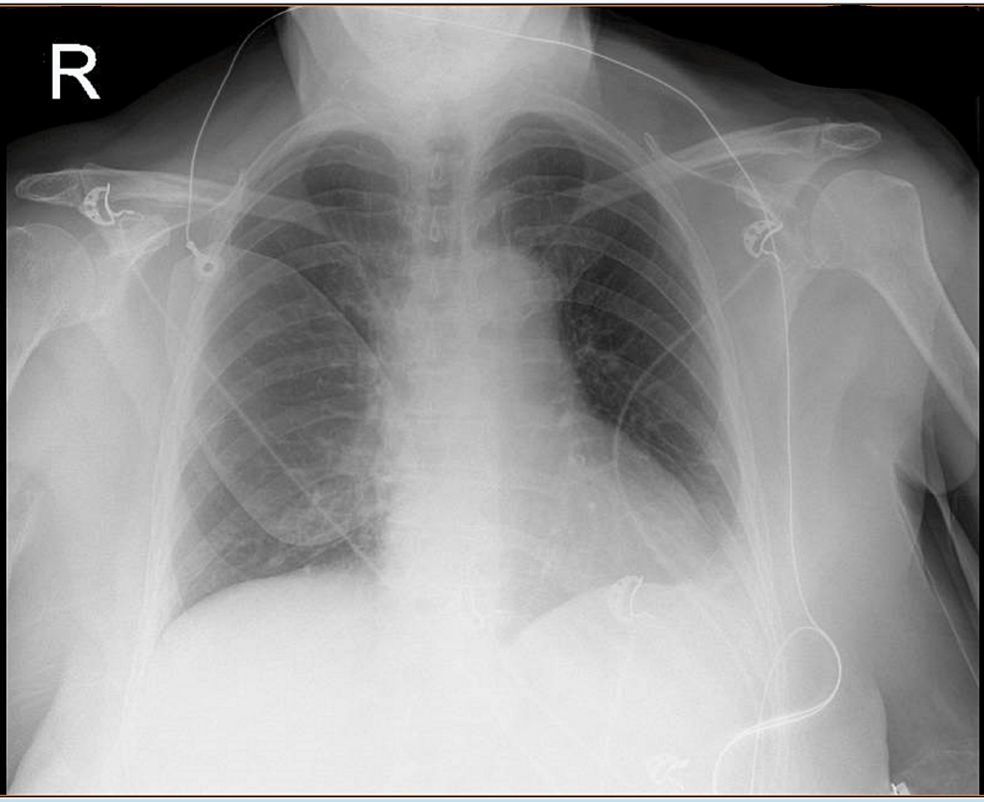
Chest X-ray Chest X-ray with defibrillator pads, no feature of pulmonary oedema, consolidation or pleural effusions.

Her chest X-ray (Figure 3) did not show any feature suggestive of pneumothorax, pulmonary oedema, pleural effusion or consolidation. After intravenous fluids and antibiotics were given, the blood results were reported.

**Figure 4:**
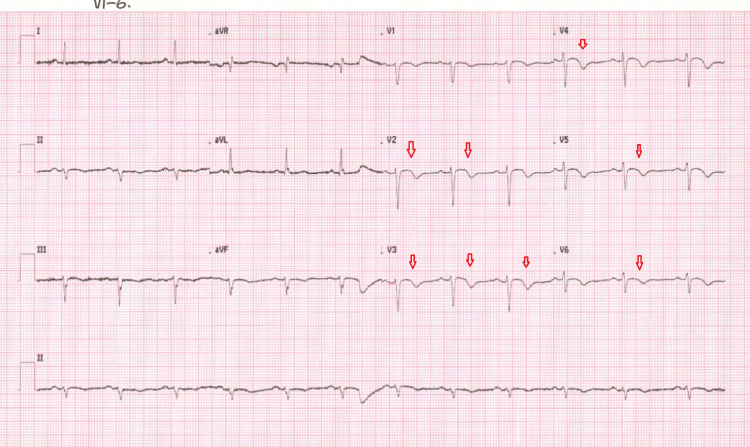
ECG ECG showed sinus rhythm with T-wave inversions in V1 to V6. ECG: electrocardiogram

Her blood results showed that her inflammatory markers were normal but her troponin results were high which was in keeping with non-ST elevation myocardial infarction (Table 2). Covid-19 RNA test was negative. She had been on treatment dose low molecular weight heparin for incidental pulmonary embolism. For the treatment of acute coronary syndrome, she was given loading doses of aspirin 300 mg and clopidogrel 300 mg, followed by 75 mg each. Her low molecular weight heparin (Dalteparin 15,000 units) was not replaced with fondaparinux as per the clinical team's decision. She had been pain-free since she was first reviewed in the emergency department. Since day one, her platelet started dropping from her baseline progressively. She was on medical treatment for non-ST elevation myocardial infarction and referred to a tertiary centre for an inpatient coronary angiogram. Given her oncology background, she received regular input from the oncology team and oncologists suspended her palliative chemotherapy which was capecitabine. On day 3 her platelet dropped further to 88 x 10^9/L and she had further chest pain. Urgent 12 lead ECG showed new T-wave inversions in antero-lateral leads (Figure 4). The case was urgently discussed with the on-call cardiology specialist registrar and referred for urgent admission to the coronary care unit. During the process, the patient was given intravenous morphine and glyceryl trinitrate spray.

**Table 2:** Blood results Full blood count, liver function test, coagulation profile, renal function and troponin. APTT: activated partial thromboplastin time; GFR: glomerular filtration rate; MDRD: Modification of Diet in Renal Disease; ALT: alanine aminotransferase

	Before admission	Day 0	Day 1	Day 2	Day 3	Day 4	Day 5	Day 6	Day 7	Day 8	Day 9	Day 10	Reference range
Basophil count (10*9/L)	0.1	0	0	0	0	0	0	0	0	0	0	0	0.0-0.1
Haematocrit (L/L)	0.44	0.442	0.419	0.42	0.43	0.42	0.4	0.38	0.43	0.37	0.37	0.407	0.36-0.46
Lymphocyte count (10*9/L)	3.2	2.9	0.9	2.9	1	1.6	1.9	2	2.3	1.9	1.6	2.7	1.0-3.0
Eosinophils (10*9/L)	0.3	0.2	0	0	0	0	0	0.1	0.2	0.2	0.1	0.1	0.0-0.5
Erythrocyte sedimentation rate (mm)													-
Neutrophil count (10*9/L)	4	2.6	13.8	6.6	9.3	11.3	14.6	10.7	8	7.5	6.5	4.6	2.0-7.0
Haemoglobin estimation (g/L)	148	146	142	139	143	142	128	124	141	120	121	134	120-150
Platelet Count (10*9/L)	246	279	132	117	88	108	124	174	252	274	277	467	150-410
Mean corpuscular volume (MCV) (fL)	102.3	100.9	98.6	101	102	102	105	104	104	105	102	100.2	83-101
Total white cell count (10*9/L)	8.4	6.3	15.7	11.2	11.2	14.2	18.5	13.9	11.4	10.6	9.3	8.2	4.0-10.0
APTT Ratio		1.3	1.2	1.02	1	1.2					1.1		0.85-1.15
Fibrinogen level (g/L)		3.68	4.01		6.18	4.64					7.03		2.00-5.10
International normalised ratio		1.1	1.2	1.31	1.2	1.7					1.6		0.90-1.10
Prothrombin Time (s)		13.2	13.4	14.5	14	20.2					19		10.20-12.60
Serum C reactive protein level (mg/L)	10	23	26						157	52	2	1	0-9
Serum troponin T level (ng/L)		123	244	566	346						39		<14
Serum Creatinine (Âµmol/L)	77	72	67	75	64	65	62	61	64	69	61	70	45-84
GFR calculated abbreviated MDRD (mL/min/1.73m*2)	62	67	73	64	77	76	80	81	77	70	81	69	-
Serum potassium (mmol/L)	4.5	4.4	4.5	4.8	4.3	4	4	4.5	4.1	4.7	4.3	4.8	3.5-5.0
Serum sodium (mmol/L)	141	141	137	142	140	139	132	132	126	130	127	131	132-146
Serum urea level (mmol/L)	3.2	3.5	4.3	3.2	3.3	3.4	3.5	4.3	7.6	6.7	5	4.1	2.5-6.7
Serum albumin (g/L)	49	46	46	42	41	44	44	42	43				35-48
Serum alkaline phosphatase (U/L)	86	78	78	66	82	84	84	81	93				30-150
Serum ALT level (U/L)	29	35	35	32	26	26	26	26	23				0-35
Serum total bilirubin level (Âµmol/L)	16	7	7		5			16	27				0-17
Serum calcium (mmol/L)	2.68	2.53	2.53	2.41	2.48	2.56	2.56	2.61					2.20-2.60
Corrected serum calcium level (mmol/L)	2.62	2.51	2.51	2.44	2.53	2.57	2.57	2.64					2.20-2.60

**Figure 5:**
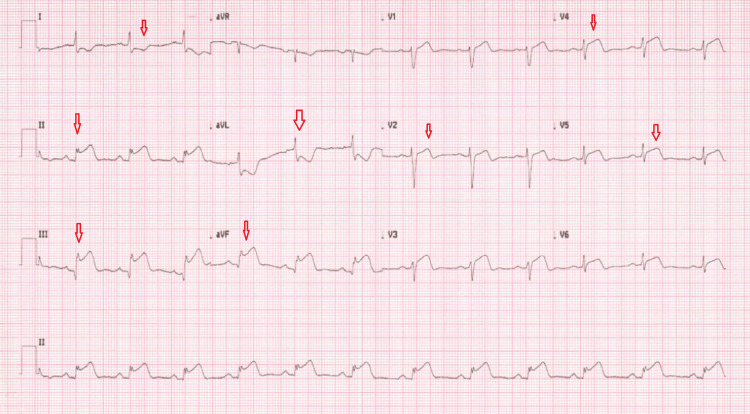
ECG ST elevation in inferior leads and anterior leads with ST depression in lead I and aVL. ECG: electrocardiogram

**Figure 6:**
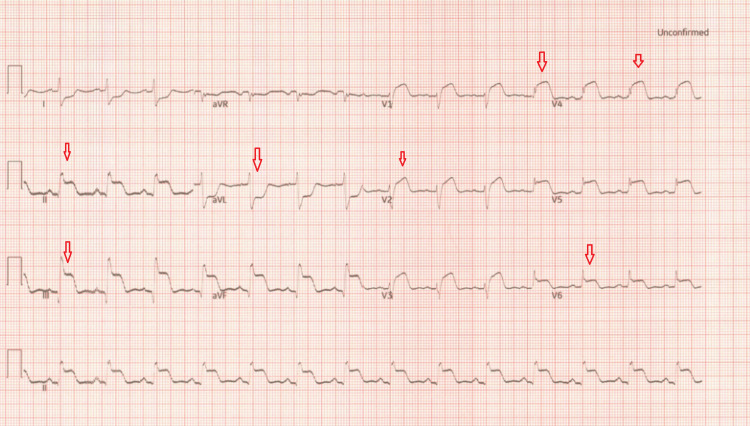
ECG ST segment in both inferior and anterior leads became more significant with deep ST depression in lead I and aVL. ECG: electrocardiogram

Just before referral, another 12 lead ECGs (Figure 5) showed highly significant ischemic changes with ST-segment elevation in inferior leads and anterior leads. Urgent primary PCI (percutaneous coronary intervention) pathway was activated and the patient was sent immediately to the cardiology centre for urgent coronary angioplasty. On arrival at the cardiology centre, repeat ECG showed widespread ST elevation in both inferior and anterior territory with ST depression in high aVL (Figure 6).

**Figure 7:**
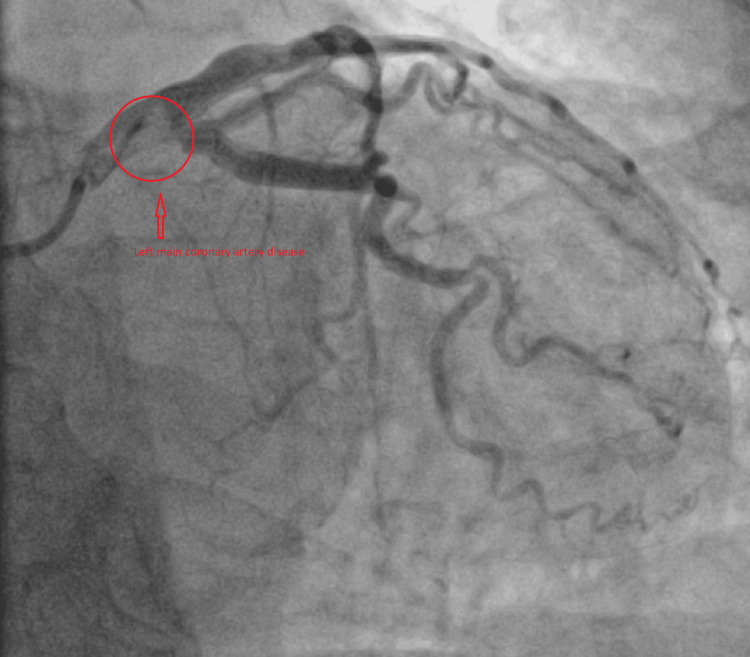
Coronary angiogram Coronary angiogram showing left main coronary artery disease.

**Figure 8:**
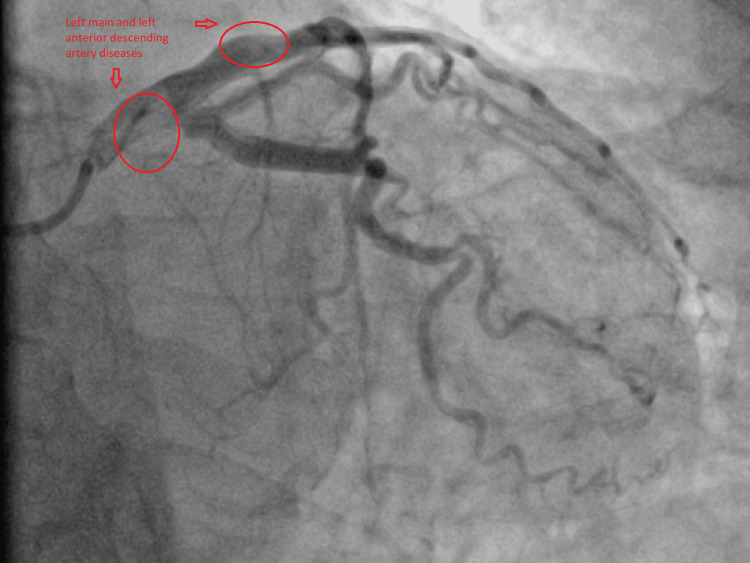
Coronary angiogram Coronary angiogram showing left main coronary artery and left anterior descending artery diseases.

**Figure 9:**
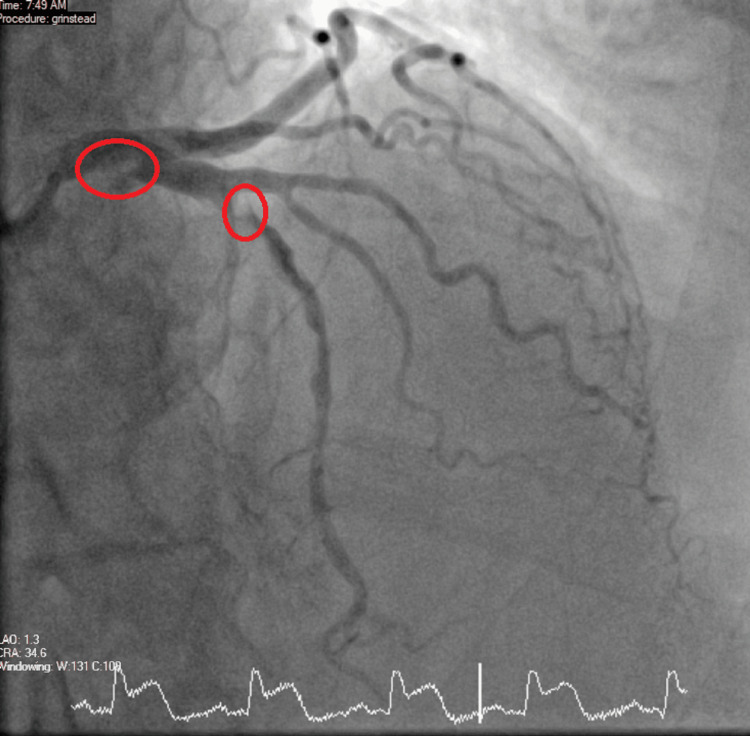
Coronary angiogram Coronary angiogram showing multiple thrombus formation in the left coronary system.

**Figure 10:**
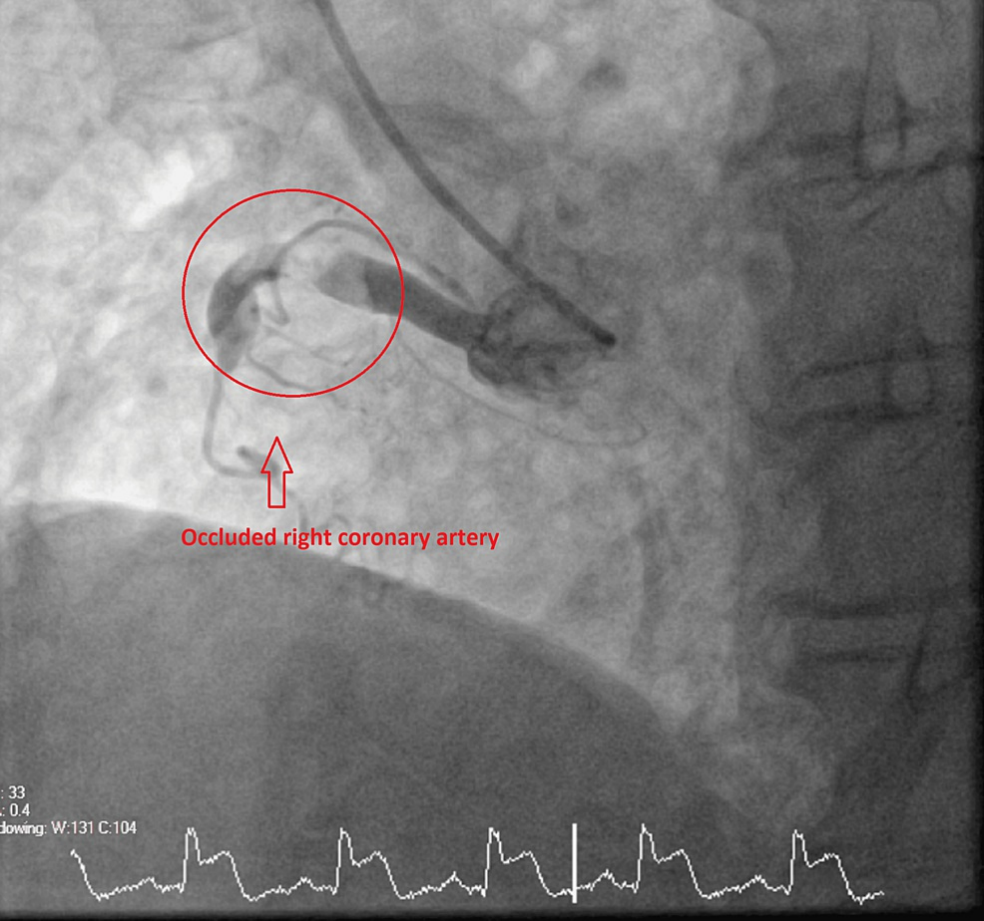
Coronary angiogram Coronary angiogram showing completely occluded right coronary artery.

The ECGs suggested that the patient may have more than one vessel territory and it was a challenge (Figures 5-6). She was immediately sent to the cardiac Cath-Lab and had a coronary angiogram. On the coronary angiogram, there was a subtotal occlusion of the right coronary artery with extensive thrombus formation and severe distal left main and left anterior descending artery disease (Figures 7-10). Despite wiring and ballooning, there was aggressive thrombus formation and the procedure was abandoned after which it was planned to be discussed in the cardiology multi-disciplinary team (MDT) for the best treatment strategy. Due to the very aggressive nature of thrombus formation in vessels, the case was reviewed by the cardiology team. The unexplained drop in platelet count was suspicious of HIT given recent treatment with low molecular weight heparin. 4T score was calculated and it scored 7 which suggested that HIT was likely. HIT screen was immediately sent and discussed with haematology. HIT screen was positive, and low molecular weight heparin was completely stopped. We started danaparoid for a total of five days followed by edoxaban as per guidance from the local haematology team. The case was discussed in a cardiology multi-disciplinary meeting to find the best revascularisation strategy. We decided to manage the patient medically than further coronary intervention. Her transthoracic echo showed severe left ventricular dysfunction with an ejection fraction of 25% with inferior, inferolateral, apex and all distal to apical wall segments were akinetic (Video 1). The right ventricle was normal and there were no valvular pathologies. Heart failure medications including ACE-I, beta-blockers and mineralocorticoid receptor antagonists were given while aspirin and edoxaban were continued. We stopped clopidogrel due to the risk of bleeding in triple therapy. Despite the major adverse cardiovascular event, she recovered well in the hospital and was discharged with follow-up plans at the pulmonary embolism clinic and cardiology clinic.

**Video 1:** Transthoracic echocardiography Parasternal long axis and apical four-chamber views showing severe left ventricular dysfunction and regional wall motion abnormalities.

## Discussion

HIT is a relatively uncommon immune-mediated phenomenon in those who have recent exposure to heparin and typically occurs within five to 10 days [9]. The pathophysiologic basis of HIT includes the formation of Ig-G antibodies against heparin/PF4 and the process leads to platelet activation and aggregation, resulting in probably increased tissue factor expression with an activated coagulation cascade. Increased platelet consumption results in a dramatic reduction in platelet count on routine blood tests.

Clinical presentations of HIT would be diverse as it might present vascular complications from thrombo-embolic events. While the lower pressure system, the venous circulation, has a greater risk for thromboembolic complications, arterial circulations can also be affected. Thrombosis in the venous system could affect both the peripheral and central venous system, including pulmonary embolism and cerebral venous sinus thrombosis [7,10]. Vascular complications in the arterial system commonly manifest as peripheral arterial systems, while central arterial thrombosis can manifest as stroke or myocardial infarction [11].

HIT is a clinical diagnosis supported by confirmatory laboratory tests. Clinical diagnosis could be based on four key criteria, which include the degree of thrombocytopenia, timing of onset, presence of thrombosis or other sequelae e.g. skin necrosis and any alternative explanation for low platelet count. These criteria are known as the "4Ts" score, and it assesses the pre-test probability of HIT. Cucker et al. in 2012 performed a meta-analysis, and it supported the robustness of the "4Ts" score in predicting HIT, and the score is also recommended by the British Society of Haematology [9,12]. In our case, the patient scored 7, which suggested that HIT was high.

The incidence of HIT in low molecular weight heparin is lower than in unfractionated heparin [3]. However, a systematic review in 2017 undertaken by Junqueira et al. concluded that evidence quality for both types of heparins was low to support the superiority of one type over the other [13].

Almeqdadi et al. summarised case reports of coronary artery thrombosis with HIT in 2018, and most cases were reported as coronaries with previous interventions. Multi-vessel thrombosis in native coronaries was reported by Ahmed et al. in 2007 [14]. Treatment strategies for acute coronary syndromes include PCI with or without thrombus aspiration [15,16]. Danaparoid and argatroban are agents recommended by the British Society of Haematology, while fondaparinux and bivalirudin could also be considered [9]. Since the presentation is not very common, typically, the diagnosis of HIT could be relatively late.

In our case, she had an incidental finding of a small volume subsegmental pulmonary embolus, and within 10 days after exposure to low molecular weight heparin, she presented with chest pain. Her other symptoms, such as lethargy, fever and diarrhoea, masked her primary pathology. When the troponin T was positive, she was being treated as a routine case of acute coronary syndrome. Her platelet count was completely normal before admission and on day one, there was nearly a fifty per cent drop in her platelet count compared to her baseline platelet. Ischemic changes on ECG presentation were also rather significant and suggested multi-vessel coronary territory involvement. On the coronary angiogram, we found that the patient had severe left main coronary artery disease, severe left anterior descending artery disease and occluded right coronary artery. Since it was an emergency, standard unfractionated heparin was used as part of the routine Cath-Lab procedure. We managed to pass the wire down into the right coronary artery and tried a non-compliant balloon. However, the extent of the thrombus was too severe and the procedure was abandoned. With a very high thrombus burden in multiple coronary vessels, the case was reviewed again and HIT was suspected. HIT laboratory test was sent and it was confirmed positive. As per the guidance from haematology, we treated her with danaparoid for five days after which we continued edoxaban. Her platelet count had always been above 100 x 10^9/L but we stopped clopidogrel given the fact that there was no cardiac stent inserted and potential bleeding risks. We continued edoxaban and aspirin for a year with a single agent (edoxaban) for life.

In our case, HIT was suspected when we see atypically high thrombus burden in multiple coronary arteries. There is no clear guidance or strategies for PCI in this group of patients as the presentation is relatively rare and might be diagnosed late. In our case, thrombus formation was significantly high and the procedure was abandoned. In the literature, we see reports of cases with successful PCIs. In our case, the patient has underlying bowel cancer which is an additional risk factor for a prothrombotic state. The case was managed at the discretion of the clinical team's expertise and experience.

Despite ST-elevation myocardial infarction, the patient recovered well and was discharged in a few days. She was followed up in cardiac rehabilitation clinic, routine cardiology clinic and pulmonary embolism clinic.

## Conclusions

As a rare entity, HIT could be a challenge to clinicians in getting the correct diagnosis in a timely fashion. Central arterial complications such as myocardial infarction could be a rare manifestation of HIT, and it can occur in patients with recent exposure to lower molecular weight heparin. The extent of thrombosis could be significantly high, and timely intervention may reduce the thrombus burden along with associated vascular complications.
